# ADHD diagnostic tools across ages: traditional and digital approaches

**DOI:** 10.3389/fpsyt.2025.1668070

**Published:** 2025-10-14

**Authors:** Marina Knyazhansky, Tammar Shrot

**Affiliations:** Department of Software Engineering, Shamoon College of Engineering, Ashdod, Israel

**Keywords:** ADHD diagnosis, virtual reality, machine learning, ecological validity, lifespan assessment

## Abstract

This article presents a narrative review of current approaches to the diagnosis of Attention-Deficit/Hyperactivity Disorder (ADHD) in children and adults. We place particular attention on recent technological advancements. ADHD diagnosis traditionally relies on a combination of subjective rating scales, clinician interviews, and observational data. In recent years, objective tools have emerged, including computerized neuropsychological tests and biometric measures. Examples include electroencephalography and eye tracking. Their clinical utility remains under investigation. This review explores these developments, including the integration of virtual reality environments and machine learning algorithms into diagnostic processes. We synthesize findings from diverse sources. The review highlights both established and emerging tools and the age-group differences in diagnostic challenges. We also note the potential of immersive and data-driven technologies to improve accuracy. Rather than applying a systematic methodology, this narrative review aims to capture current directions and preliminary insights that can inform future research hand practice. We reviewed recent research on ADHD diagnosis across age groups, with a focus on virtual reality and machine learning. We found that these tools showed modest accuracy improvements and better reflection of real-world setting, though studies were generally small and diverse. These findings suggest that VR-ML systems could develop into practical and explainable decision-support tools for everyday ADHD diagnosis.

## 1 Introduction

Attention-Deficit/Hyperactivity Disorder (ADHD) affects approximately 5 – 7% of school-aged children and 2:5% of adults worldwide. It significantly impacts education, work, and relationships. It also affects mental and physical health across the lifespan (8). The disorder is defined clinically by the Diagnostic and Statistical Manual of Mental Disorders, Fifth Edition (DSM-5), which outlines nine symptoms of inattention and nine symptoms of hyperactivity-impulsivity.

However, accurate diagnosis requires more than checklist adherence; it depends on expert clinical judgment regarding symptom chronicity, developmental appropriateness, and cross-situational functional impairment. This interpretative element, compounded by variability across sex, age, and sociocultural context, contributes to the heterogeneity of presentation and diagnostic complexity.

Despite widespread use in clinical and educational settings, conventional assessment tools have notable limitations. Behavioral rating scales, such as the Child Behavior Checklist (CBCL), Vanderbilt ADHD Diagnostic Rating Scale, and SNAP-IV, remain the cornerstone of ADHD screening and triage in primary care. Parent-report scales can achieve high diagnostic accuracy (pooled area under the curve (AUC) ≈ 0.85). However, validity is limited by inter-informant discrepancies and susceptibility to subjective bias (1). To introduce more objectivity, computerized Continuous Performance Tests (CPTs) were developed to quantify attentional lapses through omission errors, commission errors, and reaction–time variability. Meta-analyses consistently reveal only moderate sensitivity and specificity. These measures often fail to differentiate ADHD from disorders with overlapping symptoms such as anxiety or specific learning disabilities (2, 4, 29). Some commercially available systems, such as QbTest, attempt to enhance CPT paradigms by incorporating infra-red motion tracking to capture motor activity. This expansion increases the range of measurable symptoms. Although, these additions modestly improve ecological validity, diagnostic performance remains constrained, with AUC metrics hovering around 0.72 and showing inconsistent sensitivity across subdomains (2).

Parallel interest has emerged in identifying physiological and neurobiological biomarkers that could support or enhance behavioral assessments. Approaches such as electroencephalographic (EEG) monitoring of theta/beta power ratios, near-infrared spectroscopy (NIRS), and serum catecholamine profiling have shown theoretical promise. These biomarkers remain largely investigational. They are not yet reliable or practical for routine clinical use. Causes include variability in protocols, cost, and interpretability (5). Consequently, current diagnostic practices remain anchored in subjective observation and simplified laboratory tasks, which often fail to reflect the complexity of real-world attention demands.

This gap is especially problematic given the dynamic nature of ADHD symptomatology throughout life. In children, hyperactivity is typically overt, manifesting itself as motor restlessness and externalized behaviors. By contrast, in adults, hyperactivity maybe internalized and expressed through executive dysfunction, inner agitation, or impulsive decision making. In older adults, diagnostic clarity is further challenged by symptom overlap with age-associated cognitive decline and comorbid conditions such as vascular and metabolic disease. Geriatric ADHD remains markedly under-recognized. Clinicians often hesitate to prescribe stimulants due to cardiovascular concerns. With careful monitoring, pharmacologic treatment may be safe and effective in this population (7).

In response to these diagnostic challenges, recent technological advancements offer promising avenues. The convergence of virtual reality (VR) environments and machine learning (ML) techniques presents an opportunity to enhance both the ecological validity and objectivity of ADHD assessments. VR allows the simulation of everyday settings, such as noisy classrooms or busy office environments, within which CPT-like tasks can be embedded. These immersive scenarios are capable of eliciting more naturalistic attentional behaviors, while allowing researchers to systematically control environmental variables. Head-mounted displays (HMDs), when equipped with eye tracking modules, inertial measurement units (IMUs), and EEG interfaces, create data rich settings that support multi–modal behavioral capture. Early validation studies of VR-based platforms, such as Aula VR and Nesplora Aula, show convergence validity with standard CPTs. They also show modest improvements insensitivity and specificity (15, 17, 27). Importantly, these tools expand the scope of the evaluation to include not only attentional lapses, but also gaze patterns, postural control, and neuro-physiological reactivity.

The integration of ML pipelines into these environments further enhances their potential diagnostic utility. Algorithms such as support vector machines (SVMs), random forests, and neural networks, including convolutional (CNN) and recurrent (RNN) architectures, are capable of modeling complex interactions among behavioral, kinematic, and physiological features. Several recent studies have reported classification precisions that exceed 0.85 (*n* between 21 and 437) for ADHD diagnosis and subtype discrimination using fused EEG, eye tracking, and motion capture data streams (19–21, 30, 34). These findings suggest that multi–modal signal integration may offer a path toward more precise and personalized diagnostic tools, with the potential to surpass the limitations of traditional single-modality assessments.

Despite the growing interest in applying VR and ML to the diagnosis of ADHD, current literature remains fragmented. The heterogeneity justifies a narrative review. This framework allows flexible synthesis of diverse study designs, outcome types, and conceptual approaches that are not suited to meta-analysis. Most studies focus either on a narrow subset of features (e.g., behavioral or physiological alone) or explore these technologies without systematically addressing their clinical validity, generalizability, or interpretability. Moreover, there is limited synthesis of findings across age groups and modalities.

This narrative review aims to fill this gap by providing a structured analysis of empirical studies that apply VR and/or ML to ADHD diagnosis in children and adults. Specifically, we identify methodological patterns, examine the reported diagnostic performance, and propose an integrative framework that highlights current capabilities, clinical potential, and future research directions.

Although the review includes structured search and screening methods, it is framed as a narrative review due to the wide variability in study designs, outcomes, and emerging nature of the technologies involved. This approach enables a flexible synthesis of findings and conceptual trends not suited to quantitative meta-analysis.

## 2 Methods

This review adopts a narrative synthesis methodology, selected to accommodate heterogeneous study designs, diverse diagnostic technologies, and varied outcome metrics. A narrative approach allows thematic analysis of conceptual trends and methodological developments, rather than estimation of effect size.

### 2.1 Search strategy and study selection

**Figure 1:**
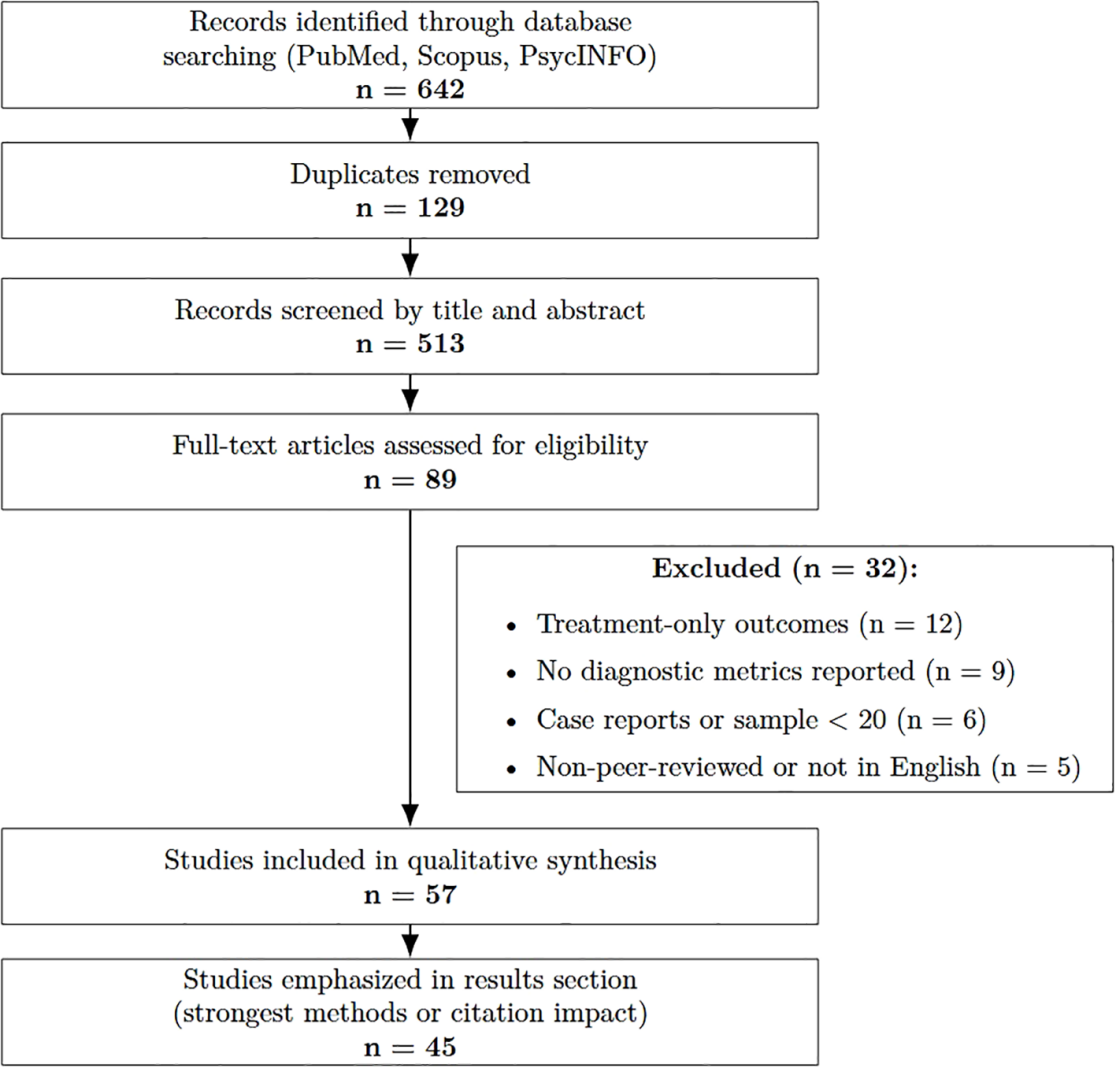
Flow diagram of the study selection process for the narrative review, adapted from PRISMA guidelines.

We mitigated selection bias by predefining inclusion/exclusion criteria, searching multiple databases (PubMed, Scopus, and PsycINFO; last search: 5 Aug 2025), and screening records in duplicate (two independent reviewers). We removed duplicates, recorded reasons for exclusion at full-text stage, and summarized the flow in a PRISMA diagram (**Figure 1**). We restricted to English language, peer-reviewed studies and did not perform a formal publication-bias analysis because no meta-analysis was conducted.

### 2.2 Data sources

We searched PubMed, Scopus, and PsycINFO for studies published 1 Jan 2014–15 May 2025 using: (ADHD OR “attention deficit hyperactivity”) AND (diagnos* OR assess* OR screen*) AND (“Continuous Performance Test” OR CPT OR “virtual reality” OR VR OR “machine learning” OR EEG OR eye tracking).

### 2.3 Eligibility

Design: Empirical evaluation of an ADHD diagnostic or screening tool

Sample: n 20 (technology development pilots excluded)

Outcomes: Quantitative diagnostic metrics (e.g., accuracy, sensitivity, specificity, AUC, F-score)

Population: Pediatric (17 y), adult (18–59 y), or older-adult (60 y) cohorts

Exclusions: case reports; non-English; non-peer-reviewed abstracts without full text; treatment only studies; plus manual snowballing of references.

### 2.4 Measures/devices

Data extracted included participant characteristics, index tests (rating scales; computerized CPTs such as QbTest/CPT-3; VR classroom/task systems with eye-tracking/EEG), feature sets, analytic models, reference standards, and psychometric indices.

### 2.5 Procedure

Titles/abstracts were screened and full texts assessed; the selection process is summarized in a PRISMA-style flow figure. Risk of bias was appraised across four QUADAS-2–adapted domains (sample selection, index test, reference standard, flow/timing).

### 2.6 Analysis

Narrative, thematic synthesis (no meta-analysis) given design/outcome heterogeneity; 57 articles met criteria, with 45 methodologically strongest or most cited emphasized. This narrative review integrates diverse evidence streams to provide preliminary insights.

**Figure 1** summarizes the search process.

## 3 Results

### 3.1 Traditional multi-informant assessments

Across nine rating-scale validation studies (*n* ≈ 3800), parent ratings, such as the CBCL, are widely used and show good diagnostic accuracy, achieved pooled AUC=0.85, while teacher ratings often lack consistency with parent reports, AUC=0.74 (1). Youth self-reports remained poorest (AUC ≈ 0.68). All questionnaire- and observation-based diagnostic methods have high ecological validity — because the person is assessed in their natural environment — but they are subjective (observer-dependent).

Risk of bias across studies was qualitatively assessed. Most studies showed moderate methodological quality, with common limitations related to small sample sizes and lack of blinding, and heterogeneity in missions and devices used.

Although computerized neuropsychological tests, such as CPTs, are popular, they are not superior to rating scales in terms of diagnostic accuracy, despite their higher cost. Executive function tests can support clinical evaluation but cannot replace traditional diagnostic methods. Objective measures, including EEG, neuroimaging, and biospecimen analysis, have shown promise in some cases but lack independent validation for clinical use. Robust time–frequency pipelines (e.g., Fourier synchrosqueezed transform combined with ICA) have been shown to improve EEG feature stability under noise and nonstationarity (40). The FDA has approved one EEG-based tool, yet its reliability remains limited, and these methods are not yet ready for widespread implementation. Research on combining rating scales through ML suggests potential improvements, with studies reporting AUC scores up to 0.98. However, comparisons between combined and single-informant assessments remain scarce, requiring further investigation. Ultimately, ADHD diagnosis depends on clinician expertise, incorporating multiple informants and standardized assessments to improve accuracy and address differential diagnoses, such as distinguishing ADHD from other mental health conditions.

### 3.2 Conventional computerized CPTs

QbTest: a CPT with motion tracking. Five studies (children *n=*682; adults *n=*514) reported moderate pooled sensitivity=0.78, specificity=0.70, AUC=0.72 (2). Sub-scales (inattention, impulsivity, activity) varied widely by age and comorbidity. QbTest is most effective when used alongside clinical assessments, as it struggles to differentiate ADHD from other clinical conditions. The tool can enhance diagnostic efficiency by reducing time to diagnosis and increasing confidence in clinician decisions, aligning with its FDA-approved and NICE recommended use. However, reliance on QbTest alone risks misdiagnosis, particularly when interpreting its subscales. Further research is needed to define its role within ADHD diagnostic pathways and validate its use across different populations.

Sendero Gris test: results indicate that the tablet-based version performs comparably to the original paper test (p=0.49), confirming its validity (3). The digital format offers advantages such as automated scoring, reduced bias, and improved accessibility. However, challenges such as data errors and small sample sizes (n=24) limit its generalizability. Despite these challenges, the study supports the digitalization of ADHD screening tools, suggesting they could improve efficiency in school based assessments. Further enhancements, including integrating additional tests and refining analysis techniques, could improve the tool’s discriminatory ability and usability.

CPT-3: evaluates attention, impulsivity, and vigilance. Studies assessing its diagnostic accuracy report moderate results, with considerable variability across subscales and study designs (4). While it effectively identifies attentional deficits and impulsivity, its sensitivity and specificity remain inconsistent, making it unreliable as a standalone diagnostic tool. CPT3 is most useful when incorporated into a broader diagnostic framework, including interviews, rating scales, and behavioral observations. Its objective data can complement clinician judgment but cannot replace traditional diagnostic methods. Standardizing cutoff scores and further research into its performance across diverse populations are necessary to refine its clinical utility. Meta-review of 11 cohorts indicated global accuracy about 0.72 but specificity dropped to 0.57 in clinical comparison groups.

da-CPT: designed to assess attention and impulsivity under realistic conditions. Compared to other ADHD diagnostic tools, such as MOXO dCPT and IVA2, it uniquely integrates auditory distractions, enhancing its ecological validity (5). The MOXOd CPT uses both visual and auditory distractors, but its emphasis on visual stimuli may not fully capture the auditory challenges ADHD individuals face, such as in noisy classrooms. In contrast, daCPT focuses exclusively on auditory distractors, making it particularly useful in assessing real-world attentional control. Compared to the IVA2, which evaluates sustained attention and impulse control through dual modality stimuli, the daCPT’s embedded auditory distractions offer a more practical simulation of everyday distractions. Studies indicate that daCPT achieves high diagnostic accuracy, particularly in detecting impulsivity and attentional lapses in distracting environments. Its ability to distinguish ADHD from non-ADHD individuals is statistically robust, making it a strong complementary tool for ADHD assessment. However, like other CPTs, it should not be used in isolation. Clinical interviews, rating scales such as Conners or Vanderbilt, and observational data remain essential for comprehensive diagnosis. Future research should explore daCPT’s role in iverse populations and its integration into multidisciplinary diagnostic frameworks. Two empirical papers embedded auditory distractors, boosting ecological validity; preliminary accuracy 0.77-0.82.

### 3.3 VR-based CPTs

Twelve studies evaluated VR paradigms. In recent developments addressing the limitations of traditional ADHD diagnostic methods, an innovative system has been introduced (16) that integrates VR, eye tracking, and EEG technologies. This new system employs VR to generate a 3D virtual classroom environment that closely mirrors real-life settings, complete with a variety of distraction factors. Within this immersive setting, subjects are evaluated using visual and auditory CPT alongside the Wisconsin Card Sorting Test (WCST), providing assessments of selective and sustained attention, abstract reasoning, and cognitive transfer abilities. The inclusion of distraction factors enables a more nuanced understanding of how external stimuli impact cognitive performance.

Aula Nesplora (HMD classroom): another VR-based CPT designed to evaluate attentional processes in children aged 6 to 16 (*n*=338). By immersing examinees in a simulated classroom environment through a VR headset, the test measures both visual and auditory attention, providing a more ecologically valid assessment than traditional computerized two-dimensional tests. This system outperformed TOVA on key attentional variables: omissions OR=3.9, commissions OR=3.1; overall AUC=0.81 (18). Head-movement variability and ocular fixation dispersion enriched predictive models (ΔAUC +0.04-0.07). Aula VR demonstrated convergent validity with Conners CPT (r=0.44-0.62) and identified latent ADHD clusters–impulsive vs. hyperactive–missed by two-dimensional indices (17). Data quality was generally high, though small sample sizes (median n=48) limited confidence intervals. Only three studies attempted machine learning fusion (e.g., gradient-boosted decision-trees) of multi-informant scales, achieving AUC up to 0.98 but lacking external validation (24).

### 3.4 Machine learning augmentation

The integration of ML techniques within VR systems has catalysed rapid advances across multiple domains, from enhancing user experience to solving practical challenges such as cybersickness and motion tracking. Recent advances in ML have been pivotal in pushing the boundaries of VR technology. Researchers have leveraged ML to enhance user experiences, detect cybersickness, predict motion intentions, and even personalize therapeutic interventions. For EEG-based pipelines, unifying temporal alignment (DTW) with low-dimensional visualization (t-SNE) has been used to interpret alpha-band EEG structures across conditions, providing interpretable embeddings of physiological features (41).

One notable contribution by Kundu et al. (9), is the development of the VR-LENS framework, which employs a super learning-based ensemble model and explainable AI (XAI) techniques to detect and classify cybersickness in VR environments. Identifying dominant features, such as eye tracking, player position, and physiological signals, the approach reduces computational complexity while maintaining high accuracy.

In the Fan et al. (10) study, a hybrid model was proposed that combines an improved AdaBoost algorithm with a long-short-term memory network (LSTM) to predict the VR user experience. This method demonstrated robust performance in classifying user experience metrics, thereby offering insight to optimize a VR system design.

Complementing these approaches, Ravva et al. (11) research on predicting upper limb motion intentions in VR-based rehabilitation has shown that multi-modal data, including eye tracking and wearable sensor input, can be effectively used to segment tasks and forecast movement directions with accuracies above 0.97. Similarly, machine learning methods have been applied to recognize user movement patterns on treadmill-like platforms, enabling more intuitive navigation within immersive VR settings (12).

An innovative study by Tang et al. (13) optimized traditional random forest classifiers through the integration of an iterative local search - sparrow search algorithm, achieving perfect classification accuracy in both the training and test sets for VR user experience prediction.

Additional research has further expanded the application of ML in VR across various domains. For instance, Wong et al. (14) conducted an open study investigating VR interventions aimed at reducing pain and anxiety in pediatric patients by tailoring immersive experiences with ML-driven personalization. This resulted in significant improvements in reported outcomes. In parallel, the study demonstrated that ML algorithms can dynamically adapt VR interfaces to enhance user engagement, suggesting that real-time personalization significantly improves interaction quality.

Eight studies harnessed ML on VR or multi-modal features (see **Table 1**).

**Table 1:** Overview of ML-enhanced VR applications: modalities, algorithms, and performance (across the papers datasets 35–146 participants, ages 6–62 years old, AUC 0.81-0.97).

Study	Modality	Algorithm	Sample (ADHD/CTL)	Accuracy/AUC	Top predictors
Wiebe et al. (19)	VR + EEG + eye	SVM	82/64 adults	0.81/0.86	omission error, theta/beta ratio, microsaccade rate
Wiguna et al. (21)	Pediatric VR game	CART	63/60 children	0.83	head-turn frequency, response-time SD
Wiguna et al. (30)	VR deep learning	CNN	40/38 children	0.91	Learnable spatiotemporal kernels
Oh et al. (20)	Multimodal fusion	Deep NN	70/65 mixed	0.89	gaze-dwell variance, EEG alpha power
Ravva et al. (11)	Motion intention	RF	36 rehab pts	0.97 (movement)	Kinematic trajectory vectors

### 3.5 Using VR with ML for ADHD

Over the past years, researchers have combined immersive VR with ML methods to build more realistic and objective tests for ADHD, moving beyond flat computer tasks into life like simulations that better reflect everyday demands (26, 27). In these VR scenarios — virtual classrooms, homes, or game worlds, participants perform typical CPTs while the system logs classic metrics (omissions, commissions, reaction-time variability) alongside new signals such as head–movement angles and eye–tracking measures (28, 29). VR allows controlled distractions (e.g., a teacher avatar or ambient noise). It exposes attentional lapses in life-like ways. Ecological validity improves compared with standard computerized 2D tests (22, 25).

ML models then learn patterns in these rich data streams. SVM trained on behavioral and motion features have distinguished adults with ADHD from controls with about 0.81 accuracy (19). Decision-tree algorithms (CART) applied to VR classroom games have accurately classified ADHD subtypes (inattentive vs. hyperactive) with over 08.83 correctness (21). Random–forest regressors using eye–movement biomarkers in a VR “treasure-hunt” game predicted standard attention and impulsivity scale scores with moderate-to-strong correlations (r ≈ 0.43 and r ≈ 0.38 (23). CNNs processing raw VR action sequences–mapping game events directly onto DSM-5 symptom criteria– have achieved above 0.90 accuracy in children (30, 31).

Combining data types further boosts performance. Deep–neural–network fusion of EEG, head kinematics, and behavioural VR features reached almost 0.89 classification accuracy, outperforming any single data stream alone (20). These multi–modal models not only improve detection but also highlight which features matter most: omission errors and head–movement variability consistently rank at the top for predicting inattention and hyperactivity (29), and gaze-dwell patterns shift noticeably when social cues are added to the VR scene (22).

Beyond pure classification, VR and ML methods can estimate symptom severity and executive function profiles in ways that mirror clinical scales. Everyday task simulations (e.g., brushing teeth, packing a backpack) yield continuous measures of prospective memory and planning. These measures align closely with parent and clinician ratings (25, 33). Survey chapters underline that these rich, multi-dimensional VR datasets are especially well suited for deep-learning, while calling for standardized VR test designs and transparent ML explainability methods (e.g. SHAP, LIME) to facilitate real-world adoption (24).

Despite these advances, studies often use small samples (many under 50 participants), and VR scenarios and feature–extraction pipelines vary widely, limiting direct comparisons (21). To move toward clinical deployment, larger multisite trials, common data sharing standards, and integrated interpretability frameworks are needed (20). After these challenges are addressed, VR-ML platforms could screen and diagnose ADHD with high accuracy. They could also personalize interventions and monitor treatment responses in naturalistic settings.

**Table 2** summarizes the diagnostic methods reviewed in this paper.

**Table 2:** Summary of the diagnostic methods reviewed in the paper: ecological validity, objectivity and accuracy (across the papers datasets 35–3800 participants, ages 6–62 years old, AUC 0.68-0.90).

Assessment method	Ecological validity	Objectivity	Accuracy/AUC (up to)
Parent Ratings	High	Low	0.84
Teacher Ratings	High	Low	0.74
Youth Self-Reports	High	Low	0.68
Combined Reporting (Parents, Teachers and Self-Reports)	High	Middle	0.86
Conventional Computerized CPTs	Low	High	0.82
VR-Based CPTs	High	High	0.81
VR-Based CPTs with ML	High	High	0.90

### 3.6 Age-specific findings

Assessment requirements differ by developmental stage. In children, primary practice is multi-informant and cross-situational — integrating parent and teacher ratings with clinical interview/observation to document impairment at home and school (35, 36). However, parent–teacher agreement is typically low to modest, which complicates threshold decisions and underscores the need for clinician synthesis (38). In adults, evaluation relies more on self-report plus collateral and evidence of functional impact at work/relationships, but confirming childhood onset is challenging because retrospective recall is often inaccurate (32).

Regarding tools, computerized/CPT-style measures and digital adjuncts can add objective data but should not replace clinical assessment. In children, adding QbTest can accelerate diagnostic decision-making without loss of accuracy (AQUA RCT) and is now recommended by NICE as an adjunct for ages 6–17; evidence is insufficient for adults (37, 39). VR-based tasks increase ecological validity by controlling distractors and capturing behavior in classroom-like contexts; studies show convergent validity and improved discrimination versus some 2D CPTs in pediatric samples (18, 27). Adult work combining VR with multimodal signals (eye-tracking/EEG/actigraphy) shows preliminary classification promise but remains early-stage and requires further validation before routine use (19).

Adults demonstrated reduced hypermotor activity but amplified executive function and emotional dysregulation signatures, discernible through VR-derived planning error metrics (6, 25). Comorbidities such as selective mutism and learning disorders further complicate developmental trajectories, underscoring the need for multimodal diagnostic strategies (43). Older adult cohorts (n=97 in two studies) showed slower head rotation recovery times and longer fixation durations, distinct from mild cognitive impairment patterns, suggesting that VR tasks can help differential diagnosis in geriatric settings (7).

**Table 3** summarizes the advantages and limitations of diagnostic methods reviewed in this paper for children and adults.

**Table 3:** Summary of the diagnostic methods reviewed in the paper: advantages and limitations for children and adults.

Assessment method	Children: advantages	Children: limitations	Adults: advantages	Adults: limitations
Traditional clinical methods (clinical interview, observation; parent/teacher ratings; in adults: self-report and collateral)	Multi-informant, cross-situational view (home/school); aligns with guideline-based assessment pathways.	Low–modest parent–teacher agreement → discrepant classifications; observer-dependence.	Validated self-report tools (e.g., ASRS) help screening/triage when combined with expert interview and collateral.	Hard to document childhood onset; retrospective recall is often inaccurate; symptom camouflage and comorbidity complicate interpretation.
Computerized tests/CPTs (incl. motion-tracking adjuncts)	Objective, standardized indices (sustained attention and impulsivity); useful adjunct to history and ratings.	Insufficient standalone diagnostic accuracy in meta-analyses; heterogeneous sensitivity and specificity.	Quantifies deficits to support differential diagnosis when interpreted clinically.	As a single test, poor predictor of ADHD; results influenced by other disorders and meds — not a replacement for clinical assessment.
Digital adjuncts (e.g., QbTest)	Speeds time-to-diagnosis without loss of accuracy in child services (AQUA RCT); NICE DG60 recommends as adjunct (ages 6–17).	Not a standalone diagnostic; service benefits depend on pathway design and training.	Evidence base thinner; may help as supportive data with interview and impairment assessment.	No routine endorsement for adults; more trials needed in adult pathways.
VR and multimodal biometrics (VR-CPT, eye-tracking, EEG, actigraphy)	Higher ecological validity (controlled distractors, classroom-like contexts); convergent validity vs. 2D CPT; some studies show better discrimination.	Small samples, heterogeneous tasks, hardware; norms, cut-offs still developing.	Early studies show multimodal VR+ML can classify adult ADHD above chance using eye-tracking, EEG, actigraphy, behavior.	Evidence in adults is preliminary; not guideline-endorsed for routine diagnosis yet.

## 4 Discussion

Our synthesis highlights the incremental yet meaningful contributions of VR and ML to ADHD diagnostics across the lifespan. While traditional CPTs offer objective metrics, they operate in low distraction, artificial environments that overlook real-world sensory-motor dynamics. In contrast, VR-based assessments place individuals in immersive, ecologically valid settings while preserving experimental control. Given the wide variability in study designs, outcomes, and technologies, a narrative synthesis was most appropriate for this review. Although structured search and appraisal methods were employed to enhance transparency, the integration of diverse findings is better suited to a narrative framework than to systematic aggregation.

Understanding why VR matters requires considering that cognitive-behavioral neuroscience posits attentional control as an embodied process intertwined with oculomotor, vestibular, and proprioceptive feedback. Head-mounted displays equipped with inertial-measurement units can quantify these subtle indices. In our review, head-movement variability consistently ranked among the top three predictors in ML models (mean SHAP value=0.19), aligning with neuroimaging reports of aberrant cerebellar-fronto-striatal connectivity in ADHD.

While ML acts as a catalyst in ADHD diagnostics, it is not a panacea. High-accuracy figures (≥ 0.90) in small-sample studies risk optimistic bias. When cross-validated across sites or devices, accuracies often decline by 0.05 – 0.1. Moreover, many pipelines lacked pre-registration, feature selection transparency and external test sets, violating TRIPOD-AI guidelines. Yet the promise is tangible: ensemble methods integrating parent ratings, VR-behavior and EEG could theoretically achieve > 0.95 AUC, approaching diagnostic gold standards in other medical specialties.

Special populations warrant attention as the scant literature on older adults underscores urgent research needs. Age-aligned normative datasets are required, as visuomotor slowing and comorbid cerebrovascular disease may confound raw metrics. Emotion-dysregulation constructs, now recognized as a possible fourth ADHD domain, should be operationalised in VR tasks by measuring affective facial micro-expressions and autonomic markers (galvanic skin response).

Several barriers to clinical adoption remain. Although the cost of HMDs and motion-capture hardware is decreasing (< USD 600 per clinic), but cybersecurity, data-privacy and device maintenance burdens persist. Implementation science frameworks (e.g., RE-AIM) suggest pilot studies embedding VR-ML assessments into pediatric and adult mental-health workflows, tracking acceptability, feasibility and cost-utility.

While performance metrics from VR and ML studies are promising, their real-world integration into clinical practice remains limited. Current ADHD diagnosis depends on clinician interviews, DSM-5 symptom mapping, and multi-informant reports. VR tools can serve as adjuncts in ambiguous cases or to enrich assessment depth. Evidence from VR-based rehabilitation in neurodevelopmental disorders supports the feasibility and ecological validity of immersive interventions, suggesting translational potential for diagnostic settings as well (44). For example, a VR classroom simulation can quantify gaze stability, motor control, and omission errors in a life like context, with ML classifiers highlighting atypical behaviour patterns. Such tools may flag individuals for further assessment or clarify subtype presentations, especially when traditional ratings conflict.

However, deploying VR + ML systems in practice presents logistical and regulatory challenges. Clinical settings must consider device availability, staff training, EHR integration, and cybersecurity. In addition, physiological-signal pipelines increasingly incorporate liveness verification steps (e.g., DTW-based checks) to curb spoofing and artefactual matches, paralleling needs in clinical assessment contexts (42). Moreover, diagnostic algorithms involving ML may fall under medical device regulations, requiring transparent validation pipelines. Embedding these tools into intake or triage processes through pilot programs could help evaluate their utility, cost-effectiveness, and clinician trust. For now, these systems should be viewed as decision-support aids, not replacements for comprehensive clinical judgment.

Beyond traditional ML approaches, deep learning (DL) methods such as convolutional and recurrent neural networks show strong potential to automatically extract complex temporal–spatial patterns from VR, EEG, and eye-tracking data. These models can capture subtle, non-linear signatures of ADHD that may be missed by simpler algorithms. However, their “black-box” nature raises barriers for clinical adoption. Embedding XAI frameworks, such as SHAP or counterfactual visualizations, into DL pipelines will be essential to bridge the gap between accuracy and interpretability, ensuring that clinicians and patients understand why a particular diagnostic suggestion was made.

An important limitation across the reviewed literature is the predominance of small sample sizes, with many studies enrolling fewer than 50 participants. Such sample constraints reduce statistical power, inflate the risk of overfitting in ML models, and limit the ability to generalize findings across diverse populations. Small, homogenous samples also make it difficult to assess performance across ADHD subtypes, sex, age groups, or comorbid conditions. As a result, even promising accuracy metrics should be interpreted cautiously until validated in larger, more representative cohorts.

## 5 Limitations of the review

This review has several limitations. First, although we applied structured search and screening procedures, the narrative synthesis approach does not provide quantitative effect estimates and is more vulnerable to selection bias than a systematic meta-analysis. Second, the included studies were highly heterogeneous in design, sample size, and diagnostic protocols, which limited the ability to make direct comparisons or pooled inferences. Third, because our review emphasizes recent technological innovations, earlier foundational work and gray literature may have been underrepresented. Finally, as a preliminary narrative review, our analysis should be considered exploratory rather than definitive, intended to highlight emerging directions rather than establish firm clinical recommendations.

To advance the field, we propose the following future research directions:

- Standardised stimuli–A core VR “classroom & office” protocol with adjustable distractor density, publicly released under open license.
- Cross-site consortia–Multi-centre trials pooling _≥ 1000_ participants to derive robust lifespan norms and to benchmark across device manufacturers.
- XAI–Mandatory deployment of model-agnostic interpretation to support clinician trust and regulatory approval.
- Deep Learning + XAI–Develop standardized pipelines where deep neural networks process multi-modal VR/biometric data, paired with model-agnostic XAI tools. This will balance predictive accuracy with transparency, enabling clinicians to validate and trust algorithmic insights.
- Hybrid decision support–Integrate VR-ML outputs with electronic-health-record (EHR) structured questionnaires, enabling automated pre-visit triage.
- Ethical governance–Frameworks addressing consent, data sovereignty and algorithmic bias, particularly for neurodiverse and minority populations.

## 6 Conclusion

Diagnostic science for ADHD is at a pivotal juncture. Rating scales remain indispensable yet intrinsically subjective; conventional CPTs quantify attention but undersample real-world complexity; and biomarker research, while promising, is still maturing. Virtual-reality assessment, especially when combined with machine learning analytics, helps bridge the gap between controlled laboratory tests and real-world conditions, capturing multisensory distraction management, motor behavior, and neurophysiological signals in a single session.

Empirical evidence to date suggest preliminary, moderate-to-large gains in diagnostic accuracy (absolute AUC improvement ≈ 0.05-0.10), improved subtype differentiation, and greater potential for personalized assessment. However, most studies remain proof-of-concept with small, non-representative samples and variable protocols. Integration into clinical work flows will require larger, multi-site trials; regulatory pathways for ML-based diagnostics; and clinician-facing explainability tools. Looking ahead, the integration of DL with XAI could provide the most powerful diagnostic augmentation, leveraging rich, multimodal VR datasets while offering interpretable outputs that align with clinical reasoning. Such approaches may ultimately allow ADHD diagnostics to combine the scalability of automated systems with the accountability required in medical contexts. VR + ML systems are unlikely to replace structured interviews or multi-informant evaluations in the near term, but they may serve as promising decision-support tools, especially in complex or ambiguous cases. Parallel advances in metacognition-oriented assessments, such as the Metacognitive Wisconsin Card Sorting Test, illustrate the translational potential of cognitive measures for developmental neuropsychology and may complement VR-ML pipelines (45).

Moving forward, efforts should focus on standardizing VR protocols, expanding normative datasets across the lifespan, and embedding these technologies into routine clinical care. Attention to older-adult populations and emotion-regulation markers will further align assessment with the lived experience of ADHD.

In sum, early-stage evidence suggests that, when used thoughtfully and ethically, VR and ML technologies have the potential to reshape ADHD diagnostics. An integrative, multimodal pathway, combining behavioral, sensorimotor and neurophysiological data within immersive tasks, may offer a more ecologically valid and personalized diagnostic process, pending larger-scale validation.

## References

[1] Peterson BS Trampush J Brown M Maglione M Bolshakova M Rozelle M. Tools for the diagnosis of ADHD in children and adolescents: a systematic review. Pediatrics. (2024) 153:e2024065854. doi: 10.1542/peds.2024-065854, PMID: 38523599

[2] Bellato A Hall CL Groom MJ Simonoff E Thapar A Hollis C. Practitioner Review: Clinical utility of the QbTest for the assessment and diagnosis of attention deficit/hyperactivity disorder – a systematic review and meta-analysis. J Child Psychol Psychiatr. (2024) 65:845–61. doi: 10.1111/jcpp.13901, PMID: 37800347

[3] Gabaldón-Pérez A Martín-Ruiz M Díez-Muñoz F Dolón-Poza M MáximoBocanegra N Pau de la Cruz I. The potential of digital screening tools for childhood ADHD in school environments: A preliminary study. Healthcare. (2023) 11:2795. doi: 10.3390/healthcare11202795, PMID: 37893869 PMC10606172

[4] Callan PD Swanberg S Weber SK Eidnes K Pope TM Shepler D. Diagnostic utility of conners continuous performance test-3 for attention deficit/hyperactivity disorder: A systematic review. J Attention Disord. (2024) 28:992–1007. doi: 10.1177/10870547231223727, PMID: 38317541

[5] Özaslan A Sevri M Ïseri E Karacan B Cengiz M Karacan H. A new objective diagnostic tool for attention-deficit hyper-activity disorder (ADHD): development of the distractor-embedded auditory continuous performance test. J Clin Med. (2024) 13:6438. doi: 10.3390/jcm13216438, PMID: 39518577 PMC11546344

[6] Soler-Gutiérrez A Pérez-González J Mayas J. Evidence of emotion dysregulation as a core symptom of adult ADHD: A systematic review. PloS One. (2023) 18:e0280131. doi: 10.1371/journal.pone.0280131, PMID: 36608036 PMC9821724

[7] Goodman DW Cortese S Faraone SV. Why is ADHD so difficult to diagnose in older adults? Expert Rev Neurother. (2024) 24:941–4. doi: 10.1080/14737175.2024.2385932, PMID: 39099142

[8] Stanford SC Sciberras E. ADHD in children and adults: diagnosis and prognosis. In: Stanford SC Sciberras E, editors. New discoveries in the behavioral neuroscience of attention deficit hyperactivity disorder. Cham, Switzerland: Springer Nature (2022). p. 1–18.

[9] Kundu RK Elsaid OY Calyam P Hoque KA. “VR-LENS: super learning-based cybersickness detection and explainable AI-guided deployment in virtual reality,” In: Proceedings of the 28th International Conference on Intelligent User Interfaces. New York, NY, USA: Association for Computing Machinery (2023). pp. 819–34.

[10] Fan W Ding Z Huang R Zhou C Zhang X. “Improved adaBoost for virtual reality experience prediction based on long short-term memory network.” In: Proceedings of the 2nd International Conference on Software Engineering and Machine Learning. Oxford, United Kingdom: EWA Publishing (2024).

[11] Ravva PU Kullu P Abrar MF Barmaki RLA. “Machine learning approach for predicting upper limb motion intentions with multimodal data in virtual reality.” In: Conference on Health, Inference, and Learning. New York, NY, USA: JMLR, Inc (2024).

[12] Sakabe FK Ayres FJ Soares LP. “Machine learning applied to locomotion in virtual reality,” In: SVR '24: Proceedings of the 26th Symposium on Virtual and Augmented Reality. New York, NY, USA: Association for Computing Machinery. (2024). pp. 134–9.

[13] Tang X Li F Cao Z Yu Q Gong Y. “Optimising random forest machine learning algorithms for user VR experience prediction based on iterative local search–sparrow search algorithm,” In: 2024 6th International Conference on Communications, Information System and Computer Engineering (CISCE), Piscataway, NJ, USA: IEEE Xplore. (2024). pp. 1387–91.

[14] Wong CL Lui MMW Choi KC. Effects of immersive virtual reality intervention on pain and anxiety among pediatric patients undergoing venipuncture: a study protocol for a randomized controlled trial. Trials. (2019) 20:1–10. doi: 10.1186/s13063-019-3303-0, PMID: 31221208 PMC6585051

[15] Mulchay C Goller H Rice W. Virtual reality-based attention test review: the nesplora aula. J Pediatr Neuropsychol. (2024) 10:243–9. doi: 10.1007/s40817-024-00161-z

[16] Lee H Li Y Yeh S-C Huang Y Wu Z Du Z. “ADHD assessment and testing system design based on virtual reality,” In: 2017 2nd International Conference on Information Technology (INCIT), (Piscataway, NJ, USA: IEEE Xplore (2018). doi: 10.1109/INCIT.2017.8257860

[17] Zulueta A Díaz-Orueta U Crespo-Eguilaz N Torrano F. Virtual reality-based assessment and rating scales in ADHD diagnosis. Psicología Educativa. (2018) 25:13–22. doi: 10.5093/psed2018a18

[18] Rodríguez C Areces D García T Cueli M González-Castro P. Comparison between two continuous performance tests for identifying ADHD: traditional vs. virtual reality. Int J Clin Health Psychol. (2018) 18:254–63. doi: 10.1016/j.ijchp.2018.06.003, PMID: 30487931 PMC6225036

[19] Wiebe A Selaskowski B Paskin M Asché L Pakos J Aslan B. Virtual reality-assisted prediction of adult ADHD based on eye tracking, EEG, actigraphy and behavioural indices: a machine learning analysis of independent training and test samples. Transl Psychiatry. (2024) 14:508. doi: 10.1038/s41398-024-03217-y, PMID: 39741130 PMC11688437

[20] Oh S Joung Y-S Chung T-M Lee J Seok BJ Kim N. Diagnosis of ADHD using virtual reality and artificial intelligence: an exploratory study of clinical applications. Front Psychiatry. (2024) 15:1383547. doi: 10.3389/fpsyt.2024.1383547, PMID: 38887727 PMC11180838

[21] Wiguna T Bahana R Dirgantoro B Minayati K Teh SD Ismail RI. Developing attention deficits/hyperactivity disorder-virtual reality diagnostic tool with machine learning for children and adolescents. Front Psychiatry. (2022) 13:984481. doi: 10.3389/fpsyt.2022.984481, PMID: 36213908 PMC9533640

[22] Eom H Kim K Lee S Hong Y-J Heo J Kim J-J. Development of virtual reality continuous performance test utilizing social cues for children and adolescents with attention-deficit/hyperactivity disorder. Cyberpsychol Behav Soc Netw. (2019) 22:198–204. doi: 10.1089/cyber.2018.0377, PMID: 30672714

[23] Yeh S-C Lin S-Y Wu EH-K Zhang K-F Xiu X Rizzo A. A virtual-reality system integrated with neuro-behavior sensing for attention-deficit/hyperactivity disorder intelligent assessment. IEEE Trans Neural Syst Rehabil Eng. (2020) 28:1899–907. doi: 10.1109/TNSRE.2020.3004545, PMID: 32746303

[24] Hernández-Capistran J Sánchez-Morales LN Alor-Hernández G Bustos-López M Sánchez-Cervantes JL. Machine and deep learning algorithms for ADHD detection: a review. In: Innovations in machine and deep learning: case studies and applications. Cham, Switzerland: Springer (2023). p. 163–91. doi: 10.1007/978-3-031-40688-1_8

[25] Jylkkä J Ritakallio L Merzon L Kangas S Kliegel M Zuber S. Assessment of goal-directed behaviour and prospective memory in adult ADHD with an online 3D video game simulating everyday tasks. Sci Rep. (2023) 13:9299. doi: 10.1038/s41598023-36351-6, PMID: 37291157 PMC10248336

[26] Areces D Rodríguez C García T Cueli M. Is an ADHD observation-scale based on DSM criteria able to predict performance in a virtual reality continuous performance test? Appl Sci. (2020) 10:2409. doi: 10.3390/app10072409

[27] Díaz-Orueta U Garcia-López C Crespo-Eguílaz N Sánchez-Carpintero R Climent G Narbona J. AULA virtual reality test as an attention measure: convergent validity with Conners’ Continuous Performance Test. Child Neuropsychol. (2014) 20:328–42. doi: 10.1080/09297049.2013.792332, PMID: 23638628

[28] Hong N Kim J-J Kwon J-H Eom H Kim E. Effect of distractors on sustained attention and hyperactivity in youth with attention deficit hyperactivity disorder using a mobile virtual reality school program. J Attention Disord. (2022) 26:358–69. doi: 10.1177/1087054720986229, PMID: 33430697

[29] Cho YJ Yum JY Kim K Shin B Eom H Hong Y-J. Evaluating attention deficit hyperactivity disorder symptoms in children and adolescents through tracked head movements in a virtual reality classroom: the effect of social cues with different sensory modalities. Front Hum Neurosci. (2022) 16:943478. doi: 10.3389/fnhum.2022.943478, PMID: 35992945 PMC9386071

[30] Wiguna T Wigantara NA Ismail RI Kaligis F Minayati K Bahana R. A four-step method for the development of an ADHD-VR digital game diagnostic tool prototype for children using a DL model. Front Psychiatry. (2020) 11:829. doi: 10.3389/fpsyt.2020.00829, PMID: 32973578 PMC7461963

[31] Ryu SH Oh S Lee S Chung T-M. “A novel approach to diagnose ADHD using virtual reality,” In: Future Data and Security Engineering: 7th International Conference, FDSE 2020, Quy Nhon, Vietnam, November 25–27, 2020, Proceedings Vol. 12669. Cham, Switzerland: Springer. (2020). pp. 260–72.

[32] Waltereit R Ehrlich S Roessner V. First-time diagnosis of ADHD in adults: challenge to retrospectively assess childhood symptoms of ADHD from long-term memory. EurChildAdolesc Psychiatry. (2023) 32:1333–5. doi: 10.1007/s00787-023-02244-2, PMID: 37286879 PMC10326145

[33] Delgado-Gómez D Sújar A Ardoy-Cuadros J Bejarano-Gómez A Aguado D Miguelez Fernandez C. Objective assessment of attention-deficit hyperactivity disorder (ADHD) using an infinite runner-based computer game: a pilot study. Brain Sci. (2020) 10:716. doi: 10.3390/brainsci10100716, PMID: 33050130 PMC7599622

[34] Andreou G Argatzopoulou A. Unraveling ADHD Through Eye-Tracking Procedures: a scoping review. J Attention Disord. (2025) 29(11):977–88. doi: 10.1177/10870547251344731, PMID: 40524599

[35] Wolraich ML Hagan JF Jr Allan C Chan E Davison D Earls M. Clinical practice guideline for the diagnosis, evaluation, and treatment of attention-deficit/hyperactivity disorder in children and adolescents. Pediatrics. (2019) 144:e20192528. doi: 10.1542/peds.2019-2528, PMID: 31570648 PMC7067282

[36] Attention deficit hyperactivity disorder: diagnosis and management. NICE guideline, NG87 (2019). Available online at: https://www.nice.org.uk/guidance/ng87 (Accessed September 13, 2019).

[37] Digital technologies for assessing attention deficit hyperactivity disorder (ADHD). NICE, Diagnostics guidance, DG60 (2024). Available online at: https://www.nice.org.uk/guidance/dg60 (Accessed October 21, 2024).

[38] Murray AL Booth T Ribeaud D Eisner M. Disagreeing about development: An analysis of parent-teacher agreement in ADHD symptom trajectories across the elementary school years. Int J Methods Psychiatr Res. (2018) 27:e1723. doi: 10.1002/mpr.1723, PMID: 29845677 PMC6877228

[39] Hollis C Hall CL Guo B James M Boadu J Groom MJ. The impact of a computerised test of attention and activity (QbTest) on diagnostic decision-making in children and young people with suspected attention deficit hyperactivity disorder: single blind randomised controlled trial. J Child Psychol Psychiatry. (2018) 59:1298–308. doi: 10.1111/jcpp.12921, PMID: 29700813 PMC6124643

[40] Gorur K Olmez E Ozer Z Cetin O. EEG-driven biometric authentication for investigation of Fourier synchrosqueezed transform-ICA robust framework. ArabianJSciEng. (2023) 48:1090110923. doi: 10.1007/s13369-023-07858-3

[41] Ozturk H Eraslan B Gorur K. Investigation of t-SNE and dynamic time warping within a unified framework forresting-state and minor analysis visual task-related EEG alpha frequency in biometric authentication: A detailed analysis. Digit Signal Process. (2025) 160:105042. doi: 10.1016/j.dsp.2024.105042

[42] Eraslan B Gorur K Temurtas F. Liveness-verified dynamic time warping-based authentication and hybrid adaptive neuro-fuzzy inference system identification for single channel diaphragmatic breathing surface electromyography biometrics. Adv Intell Syst. (2025), 2500015. doi: 10.1002/aisy.202500015

[43] Capobianco M Costa A. Selective mutism and comorbidity with specific learning disorders: evaluation and multimodal intervention in a clinical case of a female child from 7 to 11 years of age. Children (Basel). (2024) 11:746. doi: 10.3390/children11060746, PMID: 38929325 PMC11202014

[44] Capobianco M Puzzo C Di Matteo C Costa A Adriani W. Current virtual reality-based rehabilitation interventions in neuro-developmental disorders at developmental ages. Front Behav Neurosci. (2025) 18:1441615. doi: 10.3389/fnbeh.2025.1441615, PMID: 39882439 PMC11775633

[45] Granato G Manzi G DiGiulio J Puzzo C Mattera A Adriani W. Assessing executive functions and metacognition: translational potential of the Metacognitive Wisconsin Card Sorting Test for developmental neuropsychology. Front Behav Neurosci. (2025) 19:1655310. doi: 10.3389/fnbeh.2025.1655310, PMID: 40978710 PMC12443700

